# Neuropsychiatric Involvement in Juvenile-Onset Systemic Lupus Erythematosus

**DOI:** 10.1155/2018/2548142

**Published:** 2018-05-29

**Authors:** Mohammad-Amin Khajezadeh, Gholamreza Zamani, Bobak Moazzami, Zahra Nagahi, Mahdie Mousavi-Torshizi, Vahid Ziaee

**Affiliations:** ^1^Children's Medical Center, Pediatrics Center of Excellence, Tehran, Iran; ^2^Department of Pediatrics, Tehran University of Medical Sciences, Tehran, Iran; ^3^Pediatric Rheumatology Research Group, Rheumatology Research Center, Tehran University of Medical Sciences, Tehran, Iran

## Abstract

**Objective:**

Systemic lupus erythematosus (SLE) is a complex autoimmune disorder characterized by multisystem involvement, including the nervous system. In the present study, we aimed to assess neuropsychiatric manifestations in juvenile-onset systemic lupus erythematosus (JSLE) in Iran.

**Methods:**

One hundred and forty-six pediatric onset patients with SLE who had registered in our pediatric rheumatology database were evaluated prospectively and cross sectionally within 2013-2015. Data including sex, age, age at the time of diagnosis, age at the time of study, physical examination, laboratory review, and neuropsychiatric inventory were extracted from this database. Classification of neuropsychiatric JSLE was according to the 1999 American College of Rheumatology (ACR) neuropsychiatric manifestations of SLE case definitions.

**Result:**

A total number of 41 patients with neuropsychiatric symptoms were selected. The patients' average age was 12.2 years. The most common neuropsychiatric symptoms were seizures, migraine, and depression. The mean age at the onset of symptoms was 10.2 ± 3 years. Mean follow-up period was 57±34 (range: 12-120) months. From 41 SLE patients, 18 (43.9) presented symptoms at the time of diagnosis. In thirteen (31.7%) patients, neurological symptoms were developed more than 1 year after SLE diagnosis. Headache was the most common feature (13%), followed by seizure (9.5%) and chorea (3.4%). Other neurological manifestations included cranial nerve involvement (0.7%), loss of consciousness (2.7%), and impaired deep tendon reflex neuropathy (2.5%). The least common neuropsychiatric JSLE manifestation was aseptic meningitis seen in only one patient (0.7%).

**Conclusion:**

The presence of headache, mood disorders, psychosis, depression, and other neuropsychological manifestations in a patient with JSLE should prompt investigations into diagnosis of the primary nervous system involvement in order to reduce mortality and morbidity.

## 1. Introduction

Systemic lupus erythematosus (SLE) is a complex autoimmune disorder characterized by multisystem involvement, including the nervous system [1–3]. Juvenile-onset SLE (JSLE) has more aggressive clinical course in comparison with adult-onset SLE and neuropsychiatric lupus is more frequent in the JSLE. The incidence rate of neuropsychiatric symptoms among pediatric patients diagnosed with SLE is present in about 14%-75% of all cases [4–8]. Approximately 70% of children with SLE will develop nervous system manifestations in the first year after initial diagnosis [9]. These complications seem to be more severe in children compared with adults and accompanied with higher rates of morbidity and mortality [10]. Early recognition of symptoms is crucial in prevention of permanent neurological sequel and patients' quality of life. However, there is limited evidence focused on the characteristics and outcomes of nervous system involvement in JSLE. The aim of the present study was therefore to describe the incidence and features of neurologic impairment in a cohort of JSLE patient population.

## 2. Materials and Methods

In the present cross-sectional study, medical records of patients diagnosed with SLE who attended the Pediatric Rheumatology Clinic at Children's Medical Center (Pediatrics Center of Excellence affiliated to Tehran University of Medical Sciences, Tehran, Iran) were reviewed. This study was carried out between April 2013 and April 2015. Childhood-onset SLE was defined if the diagnosis was made at an age of 18 years or less. A total number of 140 patients fulfilling the revised criteria of the American College of Rheumatology (ACR) for classification of SLE were included in this study [11]. Patients were divided into 3 groups according to age at the time of study: less than 5 years, between 5 and 10 years, and more than 10 years. Patients who had been followed up less than six months were excluded from the study. The clinical details of patients such as demographic characteristics, first clinical manifestation, family history of rheumatologic diseases, laboratory findings, age at the time of diagnosis, and neurological and psychiatric manifestations of JSLE were extracted by reviewing medical records. Neuropsychiatric JSLE consisted of 19 syndromes which were used to verify the clinical manifestations in our patients including, aseptic meningitis, cerebrovascular disease (CVD), demyelinating syndrome (e.g., multiple sclerosis), headache, movement disorder, myelopathy, seizure disorder, acute confusional state, anxiety disorder, cognitive dysfunction, mood disorder, psychosis, Guillain-Barre syndrome, autonomic neuropathy, mononeuropathy (single/multiplex), myasthenia gravis, cranial neuropathy, plexopathy, and polyneuropathy.

Para-clinical data including antinuclear antibodies, anti-double-stranded DNA (anti-dsDNA), cerebrospinal fluid (CSF) analyses, electroencephalography, nerve conductions studies and electromyography, and neuroimaging studies were obtained from the medical records. Patients with secondary causes of neuropsychiatric manifestations, such as central nervous system (CNS) infection, hypertensive encephalopathy, uremia, or other central nervous system disease not related to SLE, were excluded from the study. Written informed consent was obtained from all patients or through their legal guardians. The study was approved by ethical committee of Tehran University of Medical Sciences.

The analysis of data was performed using SPSS version 19.0 software (SPSS Inc., Chicago, IL, USA). A Student's *t*-test was used for comparison of continuous variables, and Chi-square and Fisher's exact tests were used to compare categorical data. P-values less than 0.05 were considered to be statistically significant.

## 3. Result

A total number of 146 patients who had been diagnosed with JSLE, including 36 males (24.7%) and 110 females (75.3%), were enrolled in the present study. Out of these 146 patients, 25 (17.1%) cases were younger than 5 years, 59 (40.4%) were 5-10 years, and 62 (42.5%) were older than 10 years. The mean age at the onset of symptoms was 10.2 ± 3 years. Mean follow-up period was 57±34 (range: 12-120) months. Of these, 41 (28%) developed neurological disease during a median follow-up period of 6 years (range 1-20 years). From 41 JSLE patients, who had neuropsychiatric manifestations, 18 (43.9%) presented symptoms at the time of diagnosis, 10 patients (24.4%) showed neuropsychiatric symptoms during first year after diagnosis, and thirteen (31.7%) patients more than 1 year after SLE diagnosis.

Headache was the most common feature (13%), followed by seizure (9.5%) and chorea (3.4%). Other neurological manifestations included cranial nerve involvement (0.7%), loss of consciousness (2.7%), and impaired deep tendon reflex neuropathy (2.5%). The least common neuropsychiatric SLE manifestation was aseptic meningitis seen in only one patient (0.7%).

**Figure 1:**
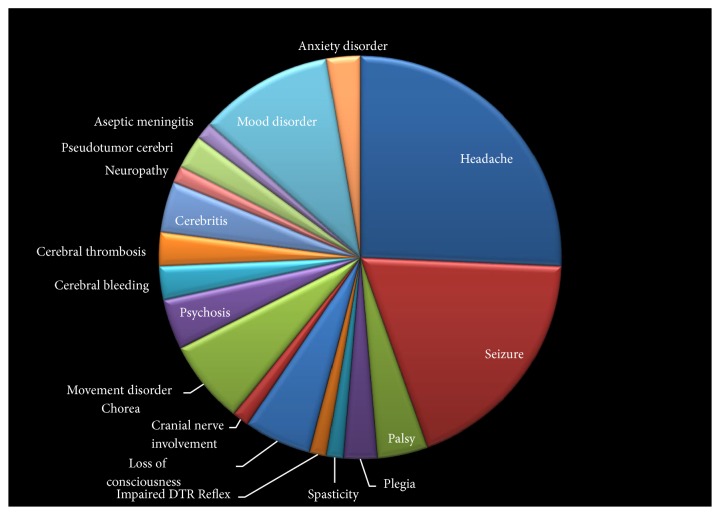
The prevalence of different neuropsychiatric manifestations of juvenile systemic lupus erythematosus.

Cognitive disorders were diagnosed in 17 patients (11.6%). Thirteen patients (8.9%) had psychiatric disorders. Among them, 3 patients (2%) experienced periods of psychosis and mood disorders were diagnosed in 8 patients (5.4%). The prevalence of other manifestations is presented in Figure 1.

In 11 patients, two different manifestations occurred simultaneously, while 6 and 2 patients experienced 3 and 4 neurological manifestations during their course of disease. Comparison between patients with and without presence of anti-dsDNA showed higher frequency of neuropsychiatric symptoms in patients with positive titer of anti-dsDNA (P < 0.05) (Table 1). No associations were found between sex and mortality with the presence of neuropsychiatric symptoms.

## 4. Discussion

Juvenile systemic lupus erythematosus (JSLE) is an autoimmune disorder with multisystem involvement, characterized by a broad spectrum of clinical features and a variable course. Although the exact underlying mechanism remains unknown, factors including environmental, hormonal, immunologic, and genetic are important contributors to the development of SLE. The occurrence of SLE is most often diagnosed in women during the second through fourth decades of life, yet the disease onset can occur at any age. Early recognition of the signs and symptoms of this complex disorder will lead to improvements in diagnosis and slowing down the debilitating effects of the disease.

More than half of pediatric patients with SLE are affected by neuropsychiatric involvement such as neurological syndromes of CNS, peripheral nervous system, and psychiatric disorders. The appearance of neuropsychiatric symptoms occurs mainly during the first year from the time of diagnosis, but it can precede the onset of SLE or occur at any time during its course [19].

Bader-Menunier et al. [12] described a median age at the onset of symptoms of 11.5 ± 2.5 years (range 1.5 to 16.0), which is similar to our study. In the present study, 43.9% of patients with neuropsychiatric JSLE developed neurological disease at the time of diagnosis, and 31.7% presented symptoms later than 1 year of diagnosis, which is in concordance with previous studies [13, 20, 21].

The female-to-male ratio in our study was 3:1, which is higher than a report by Singh et al. [13]. A higher female: male ratio has also been reported previously by Tavangar-Rad et al. [22] from Iran and Watson et al. [23] from UK.

A study done by Singh et al. [13] indicated that male patients were more likely to develop neuropsychiatric JSLE as compared to girls. On the contrary, our results did not find any association between sex and development of neuropsychiatric symptoms. A possible explanation for the observed difference could be due to differences in the ethnicities and genetic backgrounds between different studies.

The diagnosis of neuropsychiatric involvement among patients JSLE could be challenging since lupus mimics many other disorders. The most common clinical manifestations reported by various studies were headache, cognitive dysfunction, mood disturbances, and seizures [19]. The pathophysiology of neuropsychiatric JSLE is not yet fully understood. The most common neuropsychiatric manifestations observed in our study were headaches, which occurred in 13% of children. This result is similar to what has previously been reported [4, 7, 14, 15]. Seizure and mood disorders were the second and third most common manifestations (13% and 9.5%, respectively), which is lower than previously described ranges for seizure and mood disorders (13-50%) [6, 12, 14].

Seizure disorders were the second most common neuropsychiatric JSLE presentation among our patients, occurring in 9.5% of cases (Table 2). Focal seizures occurred in 2.7% of pediatric patients and generalized seizure in 6.8% (Table 2).

Mood disorders attributed to SLE were identified only in 8 (5.4%) patients in our study. It should be noted that the low prevalence of cognitive dysfunction in our patients could partly be attributed to the retrospective nature of the study for which subtle impairment of mental functions may not have been correctly identified. Major depression (4.7%), movement disorder (3.4%), psychosis (2.1%), obsessive-compulsive disorder, pseudotumor cerebri and anxiety (each one 1.4%), and involvement of peripheral nervous system (0.7%) were the other important neurological manifestations observed in our patients. These results are comparable to the 8% prevalence of mood disorders in other retrospective studies [4, 24, 25].

The prevalence of cerebrovascular disease was low in the present study, occurring in 5.3% of pediatric SLE patients. This incidence was lower compared to the 7–17% prevalence of cerebral infarction reported in the previous pediatric literatures [2, 8, 26]. While the exact mechanisms leading to cerebrovascular disease in patients with SLE are unknown, the cerebrovascular compromise seen in these patients could be secondary to focal thrombus formation as a manifestation of vasculitis [27, 28]. It has also been presumed that cerebrovascular disease in SLE could result from embolus migration to a major cerebral vessel as was documented in 2 patients in our study.

Pseudotumor cerebri is a neurological manifestation of JSLE and can be due to antiphospholipid antibodies and dural sinus thrombosis or complication of treatment after rapid reduction of steroid [29, 30]. Pseudotumor cerebri has been reported as the first manifestation of JSLE [31].

In up to 80% of our patient cohort had increased levels of serum ANA, with increased levels of anti-dsDNA values being elevated in 71.2% of patients. Serum complement levels were decreased in more than half of our patients, which is similar to other reports [7, 32]. However, serum complement levels do not seem to be a sensitive screening test for neurologic complications in JSLE. Complement deficiency was less common in patients with neurological manifestation in our previous study, although this finding was not statistically significant [33].

The diagnosis of neuropsychiatric JSLE has been hampered by the lack of sensitive and specific tests. Given that both generalized and focal nervous system are present in this disease, the concept that anti-neuronal antibodies and an immune-mediated vasculitis are likely involved in the pathogenesis of neuropsychiatric JSLE has emerged. However, no CNS specific autoantibody has been detected in either serum or CSF of these patients. Therefore, further investigation is required to determine the pathogenesis of the neural correlates of SLE in the pediatric population.

Neuropsychiatric symptoms have significant association with gastrointestinal involvements [34]. In our previous study, 33% JSLE patients with gastrointestinal manifestation had neurologic manifestations simultaneously [35].

The mortality rate in our study was 4.9% in neuropsychiatric involvement in JSLE that was more than JSLE without neuropsychiatric involvement but this difference was not statistically significant. This rate was less than other studies [34].

In a study in Polish population, STAT4 gene single-nucleotide polymorphism has been associated with increased risk of developing neurologic manifestations of SLE [36]; however, this is not confirmed in another study in Iranian children [37].

The present study has a number of limitations. First, the retrospective nature of the study underestimates the frequency of symptoms reported by patients since symptoms were only included in the chart if they were severe enough for the patient to alert the physician. Milder symptoms such as mild headache or nocturnal seizure may have not reported by patients and therefore not included in the charts. Second, mild to moderate cognitive and attention disorders could not be fully assessed in the present study since formal neurocognitive testing was not performed for all patients. Since it is more difficult for children to describe attention, mood, and cognitive symptoms, these symptoms are less likely to be identified in the present study. Third, brain MRI was not obtained in all subjects which could underestimate the prevalence of cerebrovascular disease as patients with clinically silent cerebral infarction and multifocal disease without residual neurologic deficits would not be identified. Therefore, the results of the present study should be interpreted in the context of its limitations.

In summary, this study describes the neuropsychiatric symptoms in 41 children with active neuropsychiatric JSLE. The presence of headache, mood disorders, psychosis, depression, and other neurologic manifestations in a patient with SLE should prompt investigations into diagnosis of the primary nervous system involvement in order to reduce mortality and morbidity. Development of neuropsychiatric symptoms in pediatric patients with SLE should prompt the investigation into possible diagnosis of neuropsychiatric JSLE.

## Figures and Tables

**Table 1:** Comparison between patients with and without nervous system involvement in juvenile systemic lupus erythematosus.

**Parameters**	**With neuropsychiatric** **Symptoms** **(41)**	**Without neuropsychiatric** **Symptoms** **(105)**	**P-value**
**Sex, N (%)**			
**male**	9 (22)	27 (25.7)	P > 0.05
**female**	32 (78)	78 (74.3)	P > 0.05
**Age (years)**			
**< 5**	1 (2.4)	6 (5.7)	P > 0.05
**5 – 10**	7 (17.1)	19 (18.1)	P > 0.05
**>10**	33 (80.1)	80 (76.2)	P > 0.05
**Mortality %**	2 (4.9)	4 (3.8)	P > 0.05
**ANA ≥ 1 : 1280**	37 (90.2)	90 (85.7)	P > 0.05
**Anti-dsDNA elevation**	34 (82.9)	70 (66.7)	P = 0.03^*∗*^
**Thrombocytopenia**	16 (39)	24 (22.8)	P = 0.02

ANA = anti-nuclear antibodies; dsDNA = double-stranded DNA;  ^*∗*^statistically significant.

**Table 2:** Frequency of the observed neuropsychiatric manifestations of juvenile systemic lupus erythematosus (JSLE) compared with international studies.

**Neuropsychiatric manifestations of JSLE**	**Present study ** **N= 146 **	**Parikh ** **(1995) [4]** **N= 108**	**Sibbitt (2002) [6]** **N= 130**	**Yancey (1981) [7]** **N= 37**	**Steinlin (1995) [8]** **N= 91**	**Menunier (2004) [12]** **N= 155**	**Singh (2009) [13]** **N= 53 **	**Quintero (2000) [14]** **N= 86 **	**Olfat (2004)[15]** **N= 90**	**Gheith (2017)[16]** **N= 78**	**Turkel (2001) [17]** **N= 10**	**Zambran (2014) [18]** **N= 90**
**Country**	Iran	USA	USA	USA	Canada	France	India	USA	Saudi Arabia	Egypt	USA	Colombia
**Prevalence (%)**	28.1	23.1	95	43.2	44	17	50.9	28	22.2	23.1	100	33.3
**Headache**	13	14.8	72	18.9	9.8	59.2	36.9	18.6	12.2	3.8	20	36
**Seizure disorder**	9.5	4.6	51	13.5	6.5	22.2	35.9	13.9	11.1	2.56	70	50
**Mood disorders**	5.4	8.3	57	16.2	-	29.6	9.4	-	11.1	7.69	100	36
**Psychosis**	2.1	0.9	12	8.1	12.08	-	9.4	-	-	-	70	-
**Movement disorder**	3.4	3.7	7	-	1.09	3.7	5.6	-	4.4	2.3	-	-
**Loss of consciousness**	2.7	-	35	5.4	-	-	17	4.6	5.5	2.56	-	-
**Cranial nerve involvement**	0.7	2.7	-	8.1	-	-	1.8	4.6	2.2	-	20	-
**cerebrovascular disease**	5.3	-	12	-	6.5	11.1	11.3	-	6.6	6.9	-	26
**Pseudotumor cerebri**	1.4	-	-	-	-	-	-	-	-	3.4	-	-
**Peripheral neuropathy**	0.7	-	15	2.7	-	-	5.6	-	-	1.28	20	13
**Aseptic meningitis**	0.7	-	1	-	1.09	-	3.7	-	-	-	-	-
**Palsy**	2.1	1.8	-	8.1	-	-	-	-	-	-	-	-
**Plegia**	1.4	3.7	-	-	-	-	-	-	-	-	-	-
**Spasticity**	0.7	0.9	-	-	-	-	-	-	-	-	-	-

## Data Availability

The data used to support the findings of this study are available from the corresponding author upon request.
